# A Computational Approach Identified Andrographolide as a Potential Drug for Suppressing COVID-19-Induced Cytokine Storm

**DOI:** 10.3389/fimmu.2021.648250

**Published:** 2021-06-24

**Authors:** Mohd Rehan, Firoz Ahmed, Saad M. Howladar, Mohammed Y. Refai, Hanadi M. Baeissa, Torki A. Zughaibi, Khalid Mohammed Kedwa, Mohammad Sarwar Jamal

**Affiliations:** ^1^ King Fahd Medical Research Center, King Abdulaziz University, Jeddah, Saudi Arabia; ^2^ Department of Medical Laboratory Technology, Faculty of Applied Medical Sciences, King Abdulaziz University, Jeddah, Saudi Arabia; ^3^ Department of Biochemistry, College of Science, University of Jeddah, Jeddah, Saudi Arabia; ^4^ University of Jeddah Center for Research and Product Development, University of Jeddah, Jeddah, Saudi Arabia; ^5^ Department of Biology, College of Science, University of Jeddah, Jeddah, Saudi Arabia; ^6^ Hematology Department, Regional Lab Makkah, Haddah, Saudi Arabia; ^7^ Integrative Biosciences Center, Wayne State University, Detroit, MI, United States

**Keywords:** SARS-CoV-2, COVID-19, cytokine storm, systems bioinformatics, drug-repurposing, andrographolide, TNF signaling pathway, molecular docking

## Abstract

**Background:**

The newly identified betacoronavirus SARS-CoV-2 is the causative pathogen of the coronavirus disease of 2019 (COVID-19) that killed more than 3.5 million people till now. The cytokine storm induced in severe COVID-19 patients causes hyper-inflammation, is the primary reason for respiratory and multi-organ failure and fatality. This work uses a rational computational strategy to identify the existing drug molecules to target host pathways to reduce the cytokine storm.

**Results:**

We used a “*host response signature network*” consist of 36 genes induced by SARS-CoV-2 infection and associated with cytokine storm. In order to attenuate the cytokine storm, potential drug molecules were searched against *“host response signature network”*. Our study identified that drug molecule andrographolide, naturally present in a medicinal plant *Andrographis paniculata*, has the potential to bind with crucial proteins to block the TNF-induced NFkB1 signaling pathway responsible for cytokine storm in COVID-19 patients. The molecular docking method showed the binding of andrographolide with TNF and covalent binding with NFkB1 proteins of the TNF signaling pathway.

**Conclusion:**

We used a rational computational approach to repurpose existing drugs targeting host immunomodulating pathways. Our study suggests that andrographolide could bind with TNF and NFkB1 proteins, block TNF-induced cytokine storm in COVID-19 patients, and warrant further experimental validation.

## Introduction

The ongoing pandemic by severe acute respiratory syndrome coronavirus 2 (SARS-CoV-2) infected more than 168 million people worldwide, causing more than 3.5 million deaths as of 26 May 2021 (https://www.worldometers.info/coronavirus/). SARS-CoV-2 infects the lower respiratory tract resulting in a severe respiratory disease called coronavirus disease of 2019 (COVID-19) (1). The majority of COVID-19 patients have mild symptoms, including cough, fever, body pain, which recover in a few days. However, a significant number of patients developed severe cases with difficulty in breath, respiratory and lung failure, organ damage, and even death (2–4). Accumulating studies found that an excessively high level of pro-inflammatory cytokines released, called cytokine storm, in response to SARS-CoV-2 infection triggers acute respiratory distress syndrome (ARDS) and multi-organ failure in COVID-19 patients (2, 4). The most prominent cytokines elevated in the blood plasma of severe COVID-19 patients include TNF, IL-6, IL-8, IL-10, and IL-2 (5, 6).

SARS-CoV-2 is classified as betacoronavirus closely related to two previously identified human pathogenic SARS-CoV and MERS-CoV, which caused the epidemics in 2002 and 2012, respectively (7). The SARS-CoV-2 infects the host cell with its spike protein that binds to the ACE2 receptor on the host cell surface and facilitates viral entry. SARS-CoV-2 has a plus-strand RNA genome of about 29,900 nucleotides that work as mRNA to encode viral proteins using host machinery. The genome also contains several *cis*-acting RNA elements that could be involved in viral infection and replications (8). Studies indicated that SARS-CoV-2 has evolutionary closely related to bat-derived SARS-CoV (8, 9).

Till now, no safe and effective therapy has been approved to cure COVID-19. However, life support treatment is recommended for the management and treatment of COVID-19 patients based on the symptoms and severity of the cases.

Based upon multiple clinical trial data on mild-to-severe COVID-19 patients, the US Food and Drug Administration (FDA) has approved only a single antiviral drug Veklury (remdesivir), for the treatment of COVID-19 on 22 October 2020. Furthermore, the FDA authorized eight drugs for emergency use to treat severe COVID-19 patients, and numerous other therapeutics are currently being under clinical trials (https://www.fda.gov/media/136832/download). The FDA is working with different organizations to facilitate the development of safe and effective drug molecules in combating COVID-19.

The elevated level of cytokines is the primary cause of severity in COVID-19 patients, and hence, attenuating the level of these cytokines would be a better approach to manage and treat the COVID-19 patients. Therefore, it is urgently required to identify the drug molecules for targeting the critical pathways in the human that could minimize cytokine storm in COVID-19. However, developing a new medicine is a scientifically challenging process that requires a very long time and massive money with the risk of failure. With the high rate of SARS-CoV-2 infection and the rise of new variant strains, drug repurposing became an extremely important process to identify the potential candidate drugs for treating the severe symptoms of COVID-19. Drug repurposing refers to an approach to identifying new therapeutic uses for approved or investigational drugs other than the scope of the original medical indication (10, 11). Since the repurpose drugs already passed the early-stage trial, including safety assessment, efficacy, toxicity, pharmacokinetic, pharmacodynamic, and preclinical testing, this approach has several advantages: drugs are sufficiently safe, low risk of failure, less investment, and shorten the timeline, could instantly start the tested trials in patients (11). Several drugs were repurposed against numerous diseases (12, 13). Furthermore, various computational methods, including molecular docking and dynamic simulations, have increasingly being used for structural insights into the action mechanism of existing drugs and for novel drug designing (14–18).

This study used a rational computational approach to repurpose drug molecules to minimize the cytokine storm associated with COVID-19 patients. Our previous study identified the SARS-CoV-2-mediated activation of the “*host response signature network*” responsible for cytokine storm (5). In this work, we identified andrographolide to target the “*host response signature network*” through a virtual drug screening method. Finally, we employed a structure-based molecular docking method to demonstrate that the andrographolide could bind with TNF and NFkB1, potentially blocking the TNF-mediated NFkB1 pathway underpinning cytokine storm in severe patients with COVID-19.

## Materials and Methods

### Host Regulatory Network and Biological Pathway Analysis

This study used the “*host response signature network*” identified in the human cells infected with SARS-CoV-2 in our previous study (5). Briefly, transcriptome data of SARS-CoV-2 infected normal human bronchial epithelial (NHBE) cells and controlled NHBE cells were used to identify the differentially expressed host genes (DEHGs) (19). A network was created by integrating DEHGs with human protein-protein interaction data. Furthermore, analysis of the network revealed an important sub-network of highly inter-connected 31 proteins (IL6, TNF, CXCL8, CXCL3, CXCL5, IRF9, SAA1, OAS3, CSF2, IFI6, OAS2, CSF3, IRF7, ICAM1, CXCL2, MX1, OAS1, MMP9, IL1A, IL1B, C3, TLR2, IFI27, CXCL6, CXCL1, CCL20, XAF1, IFI44L, MX2, BST2, IFITM1) predominantly involved in cytokine storm and under the regulation of transcription factors STAT1, STAT2, STAT3, POU2F2, and NFkB1. The sub-network of 31 proteins and their five master regulators are called as “*host response signature network*”. In order to understand the biological pathways associated with the “*host response signature network*”, KEGG (Kyoto Encyclopedia of Genes and Genomes) pathway enrichment analysis was performed using DAVID 6.8 (https://david.ncifcrf.gov/home.jsp).

### Potential Drugs Against “Host Response Signature Network”

The “*host response signature network*” was analyzed to find potential drug molecules using the “Gene Association” module of Network Data Exchange (NDEx version 2.4.5) (https://ndexbio.org/) (20, 21). NDEx is an open-source platform for biological network analysis and knowledge discovery. The output of “Gene Association” analysis gives networks of query gene/protein and its direct or indirect interaction with chemical and drug molecules. All output networks of drug-target interaction were merged to make a non-redundant network and visualized with Cytoscape software version 3.7. Subsequently, we analyzed the potential drug molecules targeting the “*host response signature network*”. For further study, we selected a drug molecule, andrographolide, naturally present in a medicinal plant used to treat common cold and inflammation (22).

### Data Retrieval

The three-dimensional coordinates of andrographolide were retrieved from the PubChem database with PubChem CID, 5318517. The crystal structure of human NFkB1, p50 subunit, with bound native DNA was selected and retrieved from the PDB database with PDB Id, 2V2T. The crystal structure of human TNF as a dimer with bound native inhibitor was selected and retrieved from the PDB database with PDB Id, 5MU8.

### Predicting the Drug‐Likeness and Pharmacokinetic Properties

The drug‐likeness and pharmacokinetic properties, including absorption, distribution, metabolism, excretion, and toxicity, were analyzed using the online tool “pkCSM‐pharmacokinetics” (http://biosig.unimelb.edu.au/pkcsm/). This machine learning-based method uses the various graph-based signature of chemical compounds having sets of distance patterns between atoms (23).

### Covalent Docking of Andrographolide to NFkB1

It was reported that andrographolide is a covalent inhibitor of NFkB1 and makes a covalent bond with Cys-62 of NFkB1 (24, 25). Autodock Vina (26) was used for covalent docking of andrographolide to NFkB1. To perform covalent docking, the reported atom of the andrographolide was linked to the sulfide atom of Cys-62 through a covalent bond (25). The adduct Cys-andrographolide was made flexible for rotation through various bonds using Autodock Tools (27). Then the docking of a very small molecule (e.g., water molecule) was performed, leading to the relaxed conformation of the flexible adduct Cys-andrographolide. Finally, this relaxed conformation of the adduct Cys-andrographolide was reported as the final covalent docking of andrographolide. Autodock Tools was used to prepare the protein, the ligand, and the grid box required for docking by Autodock Vina.

### Molecular Docking and Protein-Ligand Complex Analysis

For normal molecular docking, we used Dock version 6.5 (28). For TNF, the homodimer (Chains A and B) was used for molecular docking, and the native inhibitor bound within the binding site formed by both monomers (Chains A and B) was used as a probe for the TNF homodimer binding site. The protein and ligand preparation required for molecular docking and visualization at different stages of docking was performed using Chimera version 1.6.2 (29). The illustrations for binding poses were generated using Pymol version 2.4.0 (Schrödinger, LLC) and the protein-ligand interaction plots were prepared using Ligplot+ version 2.1 (30). The binding energy and dissociation constant scores were predicted using XScore version 1.2.11 (31).

## Results and Discussion

The integration and analysis of high-throughput biological data are extensively used to identify altered regulatory networks and the potential target molecules of complex diseases (5, 32). Furthermore, virtual drug screening and molecular docking were used to find therapeutic drug molecules against target molecules to treat the disease (17, 18). Therapeutic molecules against SARS-CoV-2 infection could be developed using two major approaches: (i) design the therapeutic molecules against the crucial targets of the virus (33–36); (ii) identify the disease-related gene regulatory network and then design the therapeutic molecules against it. Our previous work identified the “*host response signature network”* associated with the influx of cytokine storm through the TNF-induced NFkB1 signaling pathway (5). Our study also suggested vitamin D’s role in reducing the cytokine storm and viruses (5). In this work, we employed the later drug development strategy in which the “*host response signature network”* induced in the SARS-CoV-2 infection was used as targets to repurpose drug molecules to reduce the cytokine storm responsible for severe COVID-19.

### Biological Pathways of “Host Response Signature Network”

**Figure 1:**
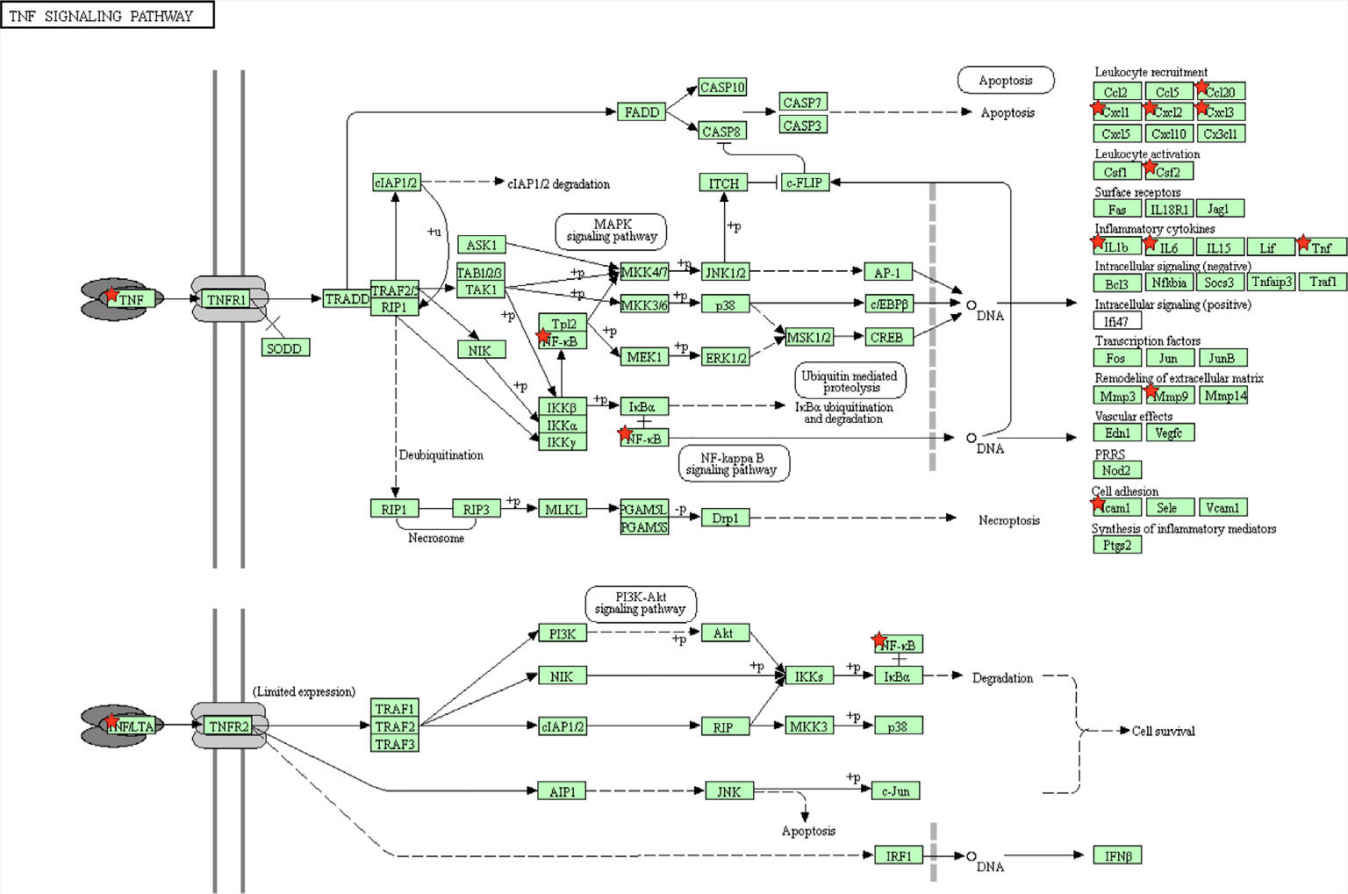
The KEGG pathway enrichment analysis showed that the *“host response signature network”* is associated with the TNF signaling pathway. The proteins IL6, CSF2, CCL20, IL1B, CXCL1, CXCL3, TNF, CXCL2, MMP9, NFKB1, and ICAM1 of *the “host response signature network”* are represented as red stars.

The “*host response signature network”* was analyzed with the KEGG pathway, which identified several significantly associated biological pathways. We found that TNF signaling pathway is significantly associated with IL6, CSF2, CCL20, IL1B, CXCL1, CXCL3, TNF, CXCL2, MMP9, NFkB1, and ICAM1 of the “*host response signature network”* and responsible for the induction of cytokine storm (**Figure 1**). The complete list of all KEGG pathways is provided in the **Supplementary Table S1**.

### Potential Drug Molecules Targeting “Host Response Signature Network”

**Figure 2:**
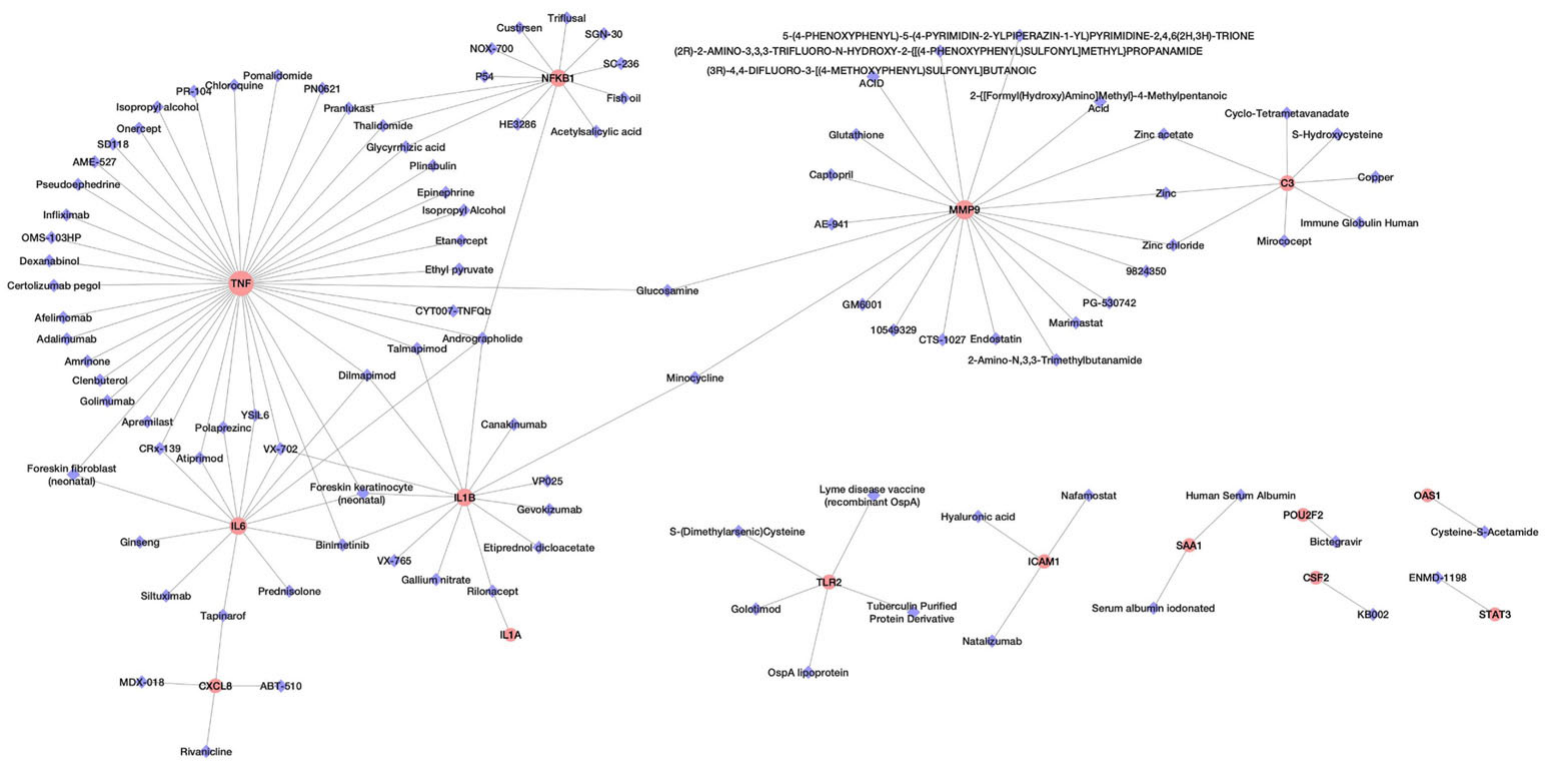
The network of drug molecules targeting the *“host response signature network”*.

Analysis of potential drug molecules for “*host response signature network*” with the “Gene Association” module of online tool NDEx returns a list of drug-target interactions networks (**Table 1**). The list of drug-target interactions from different networks was merged to create a non-redundant drug-target network (**Figure 2**). The detailed information of drug molecules is provided in the **Supplementary Table S2**. The data was further investigated to find a potential drug for better management of the cytokine storm in severe COVID-19 patients. We have selected andrographolide for further study based upon the following criteria from the list of repurposed drugs.

**Table 1:** Drug-target networks were retrieved using the “Gene Association” module of NDEx v2.4.5.

Network Name	Query result	
	Network Properties	Number of overlapping gene
DrugBank - Combined Network **Parent:** Nodes:11994; Egde:27799	Nodes:106; Egde:119	14 genes
DisGeNET - Gene-Disease Associations (Score >=0.5) **Parent:** Nodes:8631; Egde:8273	Nodes:75; Egde:79	14 genes
DrugBank - Target drugs **Parent:** Nodes:11407; Egde:19650	Nodes:104; Egde:118	13 genes
BioGRID: Protein-Chemical Interactions (H. sapiens) **Parent:** Nodes:6776; Egde:10854	Nodes: 52; Egde:45	12 genes
BindingDB - High Affinity Compounds vs. human targets (Commercially available) **Parent:** Nodes:1374; Egde:3371	Nodes: 7; Egde:6	2 genes
DrugBank - Enzyme drugs **Parent:** Nodes:1984; Egde:4905	Nodes: 2; Egde:1	1 gene

The “host response signature network” was used as a query to find the potential drug molecules.

(a) The drug must target/suppress the highest number of proteins of the “*host response signature network*”; (b) targeted proteins associate with the same biological pathway; (c) targeted proteins play a crucial role in the cytokine storm; and (d) a medicinal plant could be a source of the drug.

**Figure 3:**
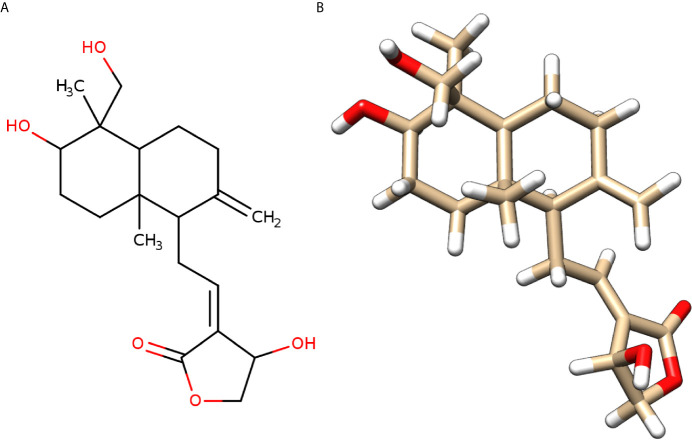
Molecular structure of andrographolide. **(A)** Two-dimensional sketch of andrographolide with O-atoms and -OH groups in red color. **(B)** Three-dimensional diagram of andrographolide with O-atoms in red and H-atoms in white colors.

We found that andrographolide can target four different proteins IL1B, NFkB1, TNF, and IL6 (**Table S2A**) of the TNF signaling pathway **(** **Figure 1** **)**, which might result in blockage of the cytokine storm in COVID-19. Andrographolide is a labdane diterpenoid isolated from *Andrographis paniculata*, also known as the “king of bitters”, a medicinal plant that mainly grows in Asian countries **(** **Figure 3** **)**. *Andrographis paniculata* traditionally used in Unani, Ayurvedic, and Chinese herbal medicines with a broad range of therapeutic applications, including treating a common cold, upper respiratory tract infections, and inflammation (22).

### Drug-Likeness and Pharmacokinetic Properties

As shown in **Table 2**, the andrographolide was having a low molecular weight of 350.46 (< 500) and the lipophilicity (LogP) value of 1.96 (<5), H-bond donors 3 (<5) and acceptors 5 (<10), and three rotatable bonds (<10). The values for all five conditions (Lipinski’s Rule of Five) for the andrographolide were well within the desired range of a drug molecule. While most of the pharmacokinetic properties, including absorption, distribution, metabolism, excretion, and toxicity, were acceptable for andrographolide (**Table 3**). Therefore, this suggests that andrographolide is a potentially safe drug candidate for use in humans.

**Table 2:** Drug-likeness (Lipinski rule of five) for andrographolide.

Lipinski rule of five		
Property	Desired value	Andrographolide
Mol Wt.	<500	350.46
H-Bond Donors	<5	3
H-Bond Acceptors	<10	5
Rotatable Bonds	<10	3
Lipophilicity (LogP)	<5	1.96

**Table 3:** Pharmacokinetic properties (ADMET) prediction for andrographolide.

Pharmacokinetic properties (ADMET)				
Property	Model Name	Desired value	Unit	Andrographolide
ABSORPTION	Water solubility		log mol/L	-3.494
	Caco2 permeability	>0.90	log Papp in 10-6 cm/s	1.07
	Intestinal absorption (human)	>>30	% Absorbed	95.357
	Skin Permeability	>-2.5	log Kp	-3.794
	P-glycoprotein substrate	No	Yes/No	No
	P-glycoprotein I inhibitor		Yes/No	No
	P-glycoprotein II inhibitor		Yes/No	No
DISTRIBUTION	VDss (human)	0.71<VDss<2.81	log L/kg	-0.286
	Fraction unbound (human)		Fu	0.281
	BBB permeability	<0.3	log BB	-0.598
	CNS permeability	>-2	log PS	-2.691
METABOLISM	CYP2D6 substrate	No	Yes/No	No
	CYP3A4 substrate	No	Yes/No	Yes
	CYP1A2 inhibitor		Yes/No	No
	CYP2C19 inhibitor		Yes/No	No
	CYP2C9 inhibitor		Yes/No	No
	CYP2D6 inhibitor		Yes/No	No
	CYP3A4 inhibitor		Yes/No	No
EXCRETION	Total Clearance		log ml/min/kg	1.183
	Renal OCT2 substrate	No	Yes/No	No
TOXICITY	AMES toxicity	No	Yes/No	No
	Max. tolerated dose (human)	<0.477	log mg/kg/day	0.128
	hERG I inhibitor	No	Yes/No	No
	hERG II inhibitor	No	Yes/No	No
	Oral Rat Acute Toxicity (LD50)		mol/kg	2.162
	Oral Rat Chronic Toxicity (LOAEL)		log mg/kg_bw/day	1
	Hepatotoxicity	No	Yes/No	No
	Skin Sensitization	No	Yes/No	No
	*T. pyriformis* toxicity	<-0.5	log ug/L	0.491
	Minnow toxicity	>-0.3	log mM	1.37

ADMET, absorption, distribution, metabolism, excretion, and toxicity; BBB, blood‐brain barrier; CNS, central nervous system; CYP, cytochrome P; hERG, human ether‐a‐go‐go‐related gene; LD50, lethal dose 50%; LOAEL, lowest observed adverse effect level; OCT2, organic cation transporter 2; VDss, steady‐state volume of distribution.

### Covalent Docking Analysis of Andrographolide With NFkB1

The PDB structures of IL1B and IL6 are available as a complex with their receptors (for example, PDB Ids: 3O4O and 1P9M, respectively) and with the antibody (for example, PDB Ids: 4G6J and 4CNI, respectively). The information for the binding site of ILIB and IL6 for a chemical compound was not obvious. So, we proceeded with the docking of andrographolide to NFkB1 and TNF only.

**Figure 4:**
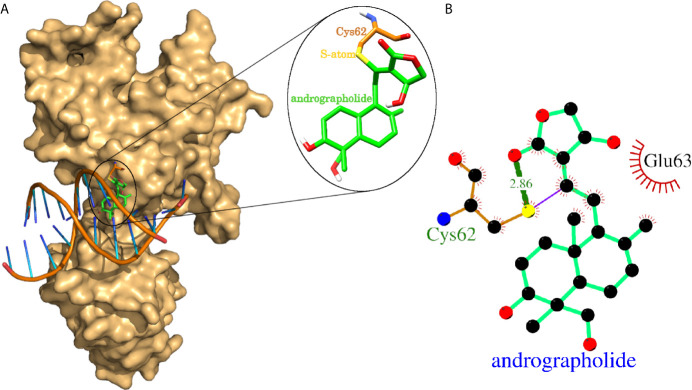
Covalent docking of andrographolide to human NFkB1. **(A)** NFkB1 is shown in surface representation colored light orange with bound native double-helical DNA and docked andrographolide. The docked andrographolide forming Cys-andrographolide adduct was shown as a close-up in the inset. The andrographolide, part of the adduct, was shown in sticks representation having carbon backbone in green color with heteroatoms oxygen, nitrogen, sulfur, and hydrogen atoms in red, blue, yellow, and white colors respectively. The Cys-62 part of the adduct forming the covalent bond through sulfur atom was also shown in sticks representation with the backbone in orange color. **(B)** Protein-ligand interaction plot of andrographolide with NFkB1. The andrographolide and hydrogen bonding residues are shown in ball and sticks representation with balls representing atoms and sticks representing the bond between two atoms. The color of balls distinguishes among atom types as C-atom in black, O-atoms in red, N-atoms in blue, and S-atom in yellow colors. The non-bonded interactions labeled with interacting residue are shown as red arcs with bristles, covalent bonds as purple line, and hydrogen bonds as thick green lines labeled with bond length in Å.

The experimental laboratories showed that andrographolide reacted with the sulfhydryl group (-SH) of Cys-62 of NFkB1 and formed a covalent bond (24, 25). One of the studies mentioned above further proceed to a normal docking of andrographolide to NFkB1, and they reported Cys-62 and Arg-57 as the interacting residues forming hydrogen bonds (25). We tried normal docking considering Cys-62 as a clue for the binding site and failed to see a binding site cavity close to Cys-62 residue where this compound may fit. Therefore, we proceeded with the covalent docking of andrographolide to NFkB1. The docking results showed that the andrographolide formed a covalent bond and a hydrogen bond (2.86 Å) with the sulfide atom of Cys-62 (**Figure 4**). The Cys-62 was also involved in 13 non-bonding contacts with andrographolide (**Table 4**). Another interacting residue Glu-63 was involved in another 13 non-bonding contacts. Thus, the andrographolide formed an adduct with Cys-62 and became part of the protein. In addition, it formed a hydrogen bond and 26 non-bonding interactions with the protein (**Figure 4** and **Table 4**). Thus, our study suggests andrographolide could block the DNA binding site, interfering with the binding of DNA, and thus the transcription factor NFkB1 would not be able to carry out its function. This is in agreement with previous findings, which revealed that andrographolide binds with NFkB1, which blocks the binding of NFkB1 to DNA and prevents transcriptional activity (24, 25).

**Table 4:** The NFkB1 residues interacting with andrographolide.

Interacting residue	Covalent bond	Hydrogen bond	Non-bonded contacts
Cys-62	1	1	13
Glu-63	–	–	13

The interacting residues are listed with a number of interactions (covalent bond, hydrogen bonds and non-bonded contacts).

### Molecular Docking Analysis of Andrographolide With TNF Homodimer

**Figure 5:**
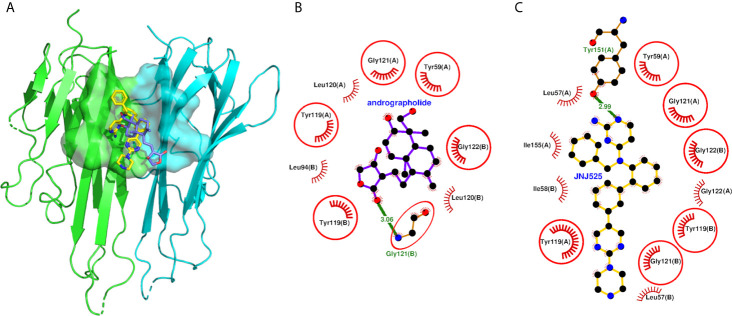
Molecular docking of andrographolide with human TNF homodimer. **(A)** The pose of andrographolide within the binding site of TNF homodimer. The NFkB1 is shown in cartoon representation colored green (Chain A) and cyan (Chain B) with the binding site in surface representation. The andrographolide and the native inhibitor are shown as sticks colored blue and yellow, respectively, with red oxygen and blue nitrogen atoms. **(B, C)** Protein-ligand interaction plots of andrographolide and the native inhibitor JNJ525 with TNF homodimer. The andrographolide and hydrogen bonding residue are shown in ball and sticks representation, with balls representing atoms and sticks representing the bond between two atoms. The color of balls distinguishes among atom types as C-atom in black, O-atoms in red, and N-atoms in blue colors. The non-bonded interactions labeled with interacting residue are shown as red arcs with bristles, while the hydrogen bond is shown as a green line labeled with bond length in Å. The residues common among interacting residues of both the native inhibitor and the andrographolide are encircled.

The andrographolide docked well within the binding site formed by two monomeric units of TNF (**Figure 5A**) and stabilized by 20 non-bonded contacts and one hydrogen bond through nine interacting residues (**Figure 5B** and **Table 5**). The absolute values of binding scores, including dock score (-32.67), binding energy (-7.52 Kcal/mol), and dissociation constant measure pK_d_ (5.51), were also reasonably high required for the stable protein-ligand complex. The nine interacting residues include four residues from chain A (Tyr-59, Tyr-119, Leu-120, and Gly-121) and five residues from chain B (Leu-94, Tyr-119, Leu-120, Gly-121, and Gly-122). Interestingly, the three identical residues Tyr-119, Leu-120, and Gly-121 from both the monomers, played a role in binding. Of nine interacting residues, Gly-121(B) formed hydrogen bonding interaction with the andrographolide with bond length 3.06 Å and also formed eight (maximum) non-bonded contacts. Therefore, the Gly-121(B) is proposed as the key residue playing a role in binding. While comparing the binding of andrographolide to that of the native inhibitor JNJ525 (**Figures 5B, C**), it was found that the andrographolide was also binding to the same site and sharing common six interacting residues Tyr-59 (A), Tyr-119 (A), Gly-121 (A), Tyr-119 (B), Gly-121 (B), and Gly-122 (B). Thus, the andrographolide was binding in the site where the TNF inhibitor is binding and inhibiting TNF activity.

**Table 5:** The residues of TNF homodimer interacting with andrographolide.

Interacting residue	No. of hydrogen bonds	No. of non-bonded contacts
Tyr-59(A)		1
Tyr-119(A)		3
Leu-120(A)		1
Gly-121(A)		1
Leu-94(B)		1
Tyr-119(B)		1
Leu-120(B)		2
Gly-121(B)	1	8
Gly-122(B)		2

The interacting residues of both chains (A and B) are listed with a number of interactions (hydrogen bonds and non-bonded contacts).

Previous studies showed that andrographolide has a potent inhibitor of wide verities of viruses, including influenza virus (37, 38), hepatitis C virus (39), herpes simplex virus type 1 (40), Chikungunya virus (41). It also has the potential to inhibit the proliferation of lung carcinoma (42) and nasopharyngeal carcinoma (43), and tumor metastasis (44). Another report demonstrated that mice treated with andrographolide reduce the inflammation and fibrosis of damaged liver (45). It also prevents liver neutrophil infiltration and reduced TNF and COX-2 signaling (45). A recent study with the molecular docking method suggested andrographolide as a potential inhibitor of SARS-CoV-2 main protease M^pro^ (47). Another computational-based study found that andrographolide and its derivative, 14-deoxy-11,12-didehydroandrographolide, have a high binding affinity with three target proteins of SARS-CoV-2, *i.e.*, main protease, papain-like protease (PL^pro^), and spike protein (48). In addition, the same study used a DIGEP-Pred tool and predicted that both compounds induce the level of several proteins involved in regulating immune systems, including the NFkB signaling pathway (48).

The NFkB1 is a family of transcription factors, which regulate the expression of various genes of pro-inflammatory cytokines, including IL6 and IL8 (CXCL8), mainly responsible for cytokine storm in COVID-19 (**Figure 1**) (5, 49). NFkB1 inhibitory protein (IkB) binds with NFkB1 dimer in an unstimulated cell, which prevents movement of NFkB1 from the cytoplasm to the nucleus. In response to SARS-CoV-2 infection, the TNF signaling pathway is activated, which causes degradation of IkB, resulting in the release and translocation of NFkB1 to the nucleus for transcription of various cytokine genes (5, 49). Our study found that andrographolide bound well with TNF and NFkB1 and provided structural insights into their binding. This binding of andrographolide blocks the TNF signaling pathways at these two critical steps, thus prevents the expression of various cytokine genes responsible for the influx of cytokine in COVID-19.

In addition, our study identified Dilmapimod as potential compounds to reduce the cytokine storm through targeting/suppressing IL1B, TNF, and IL6 (**Table S2A**). Activation of the p38 MAPK signaling pathway is associated with lung inflammation in asthma and chronic obstructive pulmonary disease (COPD) (50). The p38 MAPK regulated NFkB dependent transcription of multiple inflammatory cytokines (51). Clinical studies showed that Dilmapimod, a novel inhibitor of p38 MAPK, decreases the TNF and IL1B level in the blood of COPD patients (52, 53). Another study also suggested the potential use of Dilmapimod for COVID-19 management (54). Furthermore, our analysis identified that Prednisolone, a corticosteroid, has the potential to suppress the level of IL6 (**Table S2A**); thus, supported previous work indicating that Prednisolone could be useful in treating severe COVID-19 (55).

Previous studies showed the anti-inflammatory and anticancer effects of Dehydroxymethylepoxyquinomicin (DHMEQ), a new inhibitor of NFkB (56, 57). Another study found that DHMEQ reduces allergic airway inflammation and cytokines in a mice model of asthma (58). An investigation conducted in Jurkat T-lymphoblastic leukemia cells found that DHMEQ prevents the TNF-α-induced nuclear translocation of NFkB (46). These findings also raised the potential use of DHMEQ in inhibiting cytokine storms and thus need to be studied in more detail in treating COVID-19.

Our study used a rational computational approach to identify the potential drug molecule for COVID-19. Though it is associated with the following limitations: (i) we used the “*host response signature network*” identified in the NHBE cells infected with SARS-CoV-2 (5). The work included a limited sample size of transcriptomic data with three infected and three controlled groups (19). (ii) This study lacks *in vitro* drug screening data on human lung cells infected with SARS-CoV-2. Therefore, it is difficult to draw the effect of andrographolide on the “*host response signature network*” in SRAS-CoV-2 infected cells. (iii) Furthermore, our study lacks the experimental evaluation of the dose-dependent effect of the drug and its associated risk.

## Conclusion

The SARS-CoV-2 infection resulted in the COVID-19 pandemic killed millions of people around the world. Therefore, it is urgently needed to develop safe and effective drug molecules in a limited time to combat COVID-19. The influx of cytokines in COVID-19 patients is the prominent reason for organ damage and death. Thus, our study used the information of altered regulatory network inducing cytokine storm in COVID-19 and then repurposed andrographolide as a potential drug molecule to reduce the cytokine storm. Molecular docking analysis showed that andrographolide could inhibit NFkB1 and TNF, and thus block the pathways responsible for cytokine storm. This naturally occurring compound possesses drug-like properties and could be a promising drug for further biochemical and cell-based experimental validation for combating severe COVID-19.

## Data Availability Statement

The original contributions presented in the study are included in the article/**Supplementary Material**. Further inquiries can be directed to the corresponding authors.

## Author Contributions

FA conceived the idea, generated the data, analyzed and interpreted the results, wrote and revised the manuscript, and designed and guided the project. MR improved the idea, generated the data, analyzed and interpreted the results, wrote, and revised the manuscript. SH and MYR verified the generated data and wrote and revised the manuscript. HB and TZ verified the computational methods, wrote and revised the manuscript. KK and MJ wrote and revised the manuscript. All authors contributed to the article and approved the submitted version.

## Conflict of Interest

The authors declare that the research was conducted in the absence of any commercial or financial relationships that could be construed as a potential conflict of interest.
